# The Hospital Nursing Supplement

**Published:** 1894-09-08

**Authors:** 


"The Hospital" Nursing Mirror.

Being the Extra Nursing Supplement of "The Hospital" Newspaper.

[Contributions for this Supplement should be addressed to the Editor, The Hospital, 428, Strand, London, W.C., and should have the word "Nursing" plainly written in left-hand top corner of the envelope.]

### News from the Nursing World.

### THE MALE NURSE.

The question of the male nurse frequently comes forward, and yet no progress is made towards supplying him with systematic instruction. One member of a Board of Guardians recently petitioned for the appointment of a male nurse in an infirmary, and we should be glad to know that the plan was sanctioned. The initial difficulty of securing a suitable person once overcome, there could be no objection to establishing under him a department for the training of suitable young men. It seems a grievous thing that nothing is yet done in this matter, for whilst male patients are still in urgent need of skilled nursing, young men with a gift for the art are denied the instruction they desire. Just now the poor law infirmaries are brought more to public notice than ever before. The Press is largely concerning itself with the limited supply of trained and the incompetency of untrained workhouse nurses. Untrained men are no more to be accounted "nurses" than untrained women are, and therefore the former have a right to demand the same instruction as their sisters. At the Seamen's Hospital at Greenwich many convalescent patients show an extraordinary aptitude for tending their sick comrades, and sometimes express a wish to make nursing their profession, but their aspirations cannot be gratified because they are met with the statement, "There is no training school for men in England."

### HOLIDAYS AND THE WEATHER.

Many must have been the disappointed amongst nurses who were holiday seekers in August. Cold and gloom was so universal there was no escaping it. In England, in France, and in Switzerland and Germany came complaints of bad weather. Strange to say, in Austro-Hungary complaints of another kind have reached us. In Vienna the heat has been tropical, the streets presenting quite a deserted appearance. Buda-Pesth is still said to be very hot, and if the weather continues the same, some of the medical visitors may be able to return with tales of summer heat quite without our experience this year. Some of the intending visitors have given up attending the Congress, for fear of encountering quarantine somewhere on the route.

### THE COLONY OF BETHEL.

The colony for epileptics at Bielefeld, in Germany, with its 1,300 inhabitants, has a subject of special interest to us in England where we have no institution of the kind. We have read of the colony probably without having any idea of its aspect and surroundings, and can form no picture in our minds of a place so bright and pleasing in appearance as Bielefeld in connection with so sad a subject as epilepsy. The *Sunday at Home* for September gives a pleasant view of the little colony, its substantial buildings placed amongst trees, some of them on a gentle slope of wooded hills. As we all probably know, all possible employment is given to the inmates, only quite hopelessly bad cases coming under the care of the deacons and deaconesses as patients.

### THE WORKHOUSE NURSING ASSOCIATION.

The Workhouse Nursing Association is doing a work of incalculable public benefit. Moreover, the rules and regulations are framed in so wise a manner that only the most unlooked for misapprehension as to their purport can prevent their working to the satisfaction and benefit of the institutions on whose behalf they have been framed. The association must not therefore be discouraged by an isolated misunderstanding, such as that at the Bedford Poor Law Infirmary. Having seen the complete set of reports and correspondence on the subject of Nurse Harcourt's dismissal by the Bedford Board of Guardians, there can be no doubt that the dismissal was caused by the Visiting Committee accepting the view of one of their members who erroneously maintained that the Workhouse Nursing Association desired to secure powers of interference such as they have always repudiated. The Board fell into the same error by accepting the report of the Visiting Committee without question, and requested the nurse, in the event of her remaining at the infirmary, to withdraw from the association. This she had not under any circumstance the power to do, being bound by contract for a certain number of years to serve under the association. The association naturally inquired the cause of the nurse's dismissal, and very soon convinced the Guardians of this mistake, but unfortunately Nurse Harcourt has not been reinstated, although the Workhouse Nursing Association and the Guardians have been cleared from all imputation of *mala fides*. We hope the common sense of the Guardians, backed up by public opinion in Bedford, will not only prevent any recurrence of proceedings of this kind in future, but will secure the re-engagement of Nurse Harcourt, otherwise the Bedford Board of Guardians may find themselves classed as belonging to the least efficient type of poor law administrators.

### IRISH HUMOUR.

The *Enniscorthy Guardian* of the 25th ult. contains the most amusing account of the election of a nurse which we have ever seen in print. The proceedings took place at the County Wexford Infirmary (46 beds), at a special meeting of the "governors and governesses" of the institution, which was largely attended. It appears that the majority of the patients are Roman Catholics, that the infirmary employs two nurses, one of whom was a Roman Catholic, and the advertisement for the second invited candidates with their certificates and testimonials to attend on a certain day. Only one candidate, Miss Richards, put in an appearance, and it is implied that she did not bring her certificate owing to the staff of the City of Dublin Hospital being away on their holidays. It had been mooted abroad that Miss Richards was a Protestant, and after more than one speaker had deprecated the introduction of the religious element into the proceedings, a long discussion took place on this vexed question, in the course of which the presence of Miss Richards appears to have been forgotten, as she was never apparently invited into the room or asked to produce her papers and testimonials. Father Hore maintained that there were a great many qualified nurses in Wexford, and they had not been in any of the training establishments. Not that this interfered with their capacity as nurse, for, as a matter of fact, they were as capable as those from any training establishment. He further objected to the appointment of a certificated nurse on local grounds. The election of Miss Richards having been duly proposed and seconded, an amendment was moved by the Catholics to postpone the election for a fortnight. The amendment was lost, and Miss Richards appears to have been ultimately appointed. Thereupon Father Hore explained that he should not be taken as having any objection to "poor Miss Richards"; and before Mr. Timpson left the room he entered strong and repeated protests against the action of the governors. Terrible threats were made as to the effect of this action upon the income of the institution, but it would appear that the outside sum derived from public subscriptions cannot exceed £130 per annum.

### A FAIRY CAKE.

A so-called "fairy cake" has been playing quite a prominent part at the Lyme Regis Cottage Hospital, and the matron has written to tell us all about it, because she thinks it may act as a suggestion to those in search of an entertainment not unassociated with profit for their institution. Accompanying the letter came a photograph of the cake, quite an imposing erection, as anyone can see for themselves if they care to send eighteenpence to the matron for a copy of the photograph. The "fairy cake" is formed of three hollow round boxes, one on top of the other, each in graduated sizes, and trimmed with pink and white paper. At the top is seated a fairy doll. The cakes are filled with so-called "surprise packets," which are reached by means of a hole at the back of each cake. The company who flocked to the entertainment at the little hospital paid a small sum for the privilege of securing a treasure, and there were other original methods besides which provoked curiosity and secured pennies for the hospital. The day was dull, but visitors had the longer opportunity of increasing their acquaintance with the little hospital.

### NURSING AT VANCOUVER.

Since we last heard from Sister Francis at Vancouver, the little community have suffered a great loss in the death of their Bishop, who gave Sister Francis much encouragement and sympathy. Naturally the new appointment is looked for with some anxiety. Much depends on support from headquarters. The little Indian Hospital is entirely diocesan, and only strong assistance from the Bishop can command enough interest to secure sufficient funds to keep it going. At St. Luke's Home, we are told, they have been fairly busy. Sister Francis has now eight nurses under her, and it is a recognition of good work that the Jubilee Hospital, Victoria, now send their nurses to St. Luke's for obstetric training. The Home has a very good record in this branch of nursing, no deaths having occurred. The first case of abdominal section in Vancouver was brought thither, and did well. Whilst the actual nursing affairs are progressing well, the surrounding country was flooded for about six weeks, cutting off communication from Eastern Canada, and causing much inconvenience and disaster. The loss of the Bishop and several other valued friends cast a gloom over St. Luke's, which the bright sunshine and profusion of flowers which are now reported will, we hope, assist to dispel.

### ANOTHER SHAM NURSE.

The adoption of the nursing garb by lay women seems quite to have assumed the form of an epidemic at the present time, and no class appears free from the infection. The latest case we have heard of is that of a woman who has been representing herself as a King's College nurse for begging purposes. She represented herself as being engaged, with other nurses, in making a collection for a poor widow, the mother of eight children, now lying ill in the hospital. On the day upon which she was arrested she had obtained upwards of £6 in a poor neighbourhood in response to her pitiful story.

### NEWS FROM LABRADOR.

Our readers will remember the interesting news from Labrador which we published recently in our pages. Since then the little hospital at Indian Harbour has run the risk of destruction. Constructing is no easy matter out there, and chimneys were found impossible, consequently iron pipes had to be used. One morning a fire was lighted according to orders, and the workmen left the premises, leaving everything satisfactory. Just as the doctor and others were leaving the *Albert*, in which ship they were still residing, a crowd rushing towards the hospital attracted their attention, and on landing they found the room in which the fire had been kindled in flames. In spite of the serious danger the hospital was in, assistance was so prompt that the fire was extinguished and little harm done. On the afternoon of the same day the first patient was admitted.

### SHORT ITEMS.

In an article on "Profit-sharing in South London" which has recently appeared in a contemporary, the pension scheme put forward in January last by the Guy's Hospital Trained Nurses' Institution is especially mentioned. This fact is of interest as showing that "profit-sharing" in the nursing profession is meeting with due recognition from a financial as well as from an institutional and philanthropic point of view.—The fund for the Nurses' Home in connection with the Blackburn and East Lancashire Infirmary is the richer by the sum of £50, the result of some sports lately got up by the Blackburn Cycling Club.—A District Nursing Association has been formed amongst the four parishes of Kinguosie, Insh, Alvil, and Laggan. A trained and certificated nurse is to be appointed.—The Guardians of the Bridgend Union have appointed Miss Jane Lewis to the post of Nurse and Assistant Matron at the Bridgend Workhouse.—The Sick Aid and Nursing Society, St. Annes-on-the-Sea, has just issued its annual report. Fifty cases have been dealt with since the inauguration of the society some eighteen months since.

### Diet in Disease.

By Mrs. ERNEST HART.

SERIES II.

### I.—RICKETS.

RICKETS was at one time thought to be a disease of the bones. It is now known to be a general disease caused by malnutrition, and which is almost always preventible. The well-known changes which take place in the bones are but the signs and symptoms of a constitutional condition. Rickets in the child is the incontrovertible sign of ignorance, neglect, or incompetence in the mother or nurse. A mother should be as ashamed of her child having rickets as of its having vermin. Both mean neglect of maternal duties. The neglect may be, it is true, due to ignorance; but in these days of enlightenment and education, ignorance on matters of vital importance is inexcusable. In these days, however, of patent foods for infants ignorance shelters itself behind assumed knowledge, and patent foods plus ignorance is the fruitful source of much rickets.

A story will illustrate my meaning. Some time ago I was interested in a "bonnie baby," the only and posthumous child of a young widowed mother. The child was the joy of her heart, and its evident health and its ceaseless activity and gaiety were sources of pride and pleasure to her. She suckled the child herself. When the baby was about six months old I lost sight of it for eight months. When I saw it again I was immediately struck by its altered appearance. It was pale and peaky; it had lost its gaiety and activity, and it had a look of premature age and weariness. "Your baby is starved," I said with brutal frankness to the mother; "what are you feeding it on?" "I suckled it until it was eleven months old," she replied, "and since then I have fed it on ——," mentioning a patent food. "What is it made of?" I asked. "I don't know," was the answer. "Don't know!" I exclaimed, "don't know what you are feeding your baby on! You have only one thing to do—to bring up that baby—and you are steadily starving it into rickets by not taking the trouble to learn how to feed it properly." I presented the alarmed mother with various text-books giving the required information how to feed infants, and I hope she has learnt; otherwise her child will have rickets. This is an example of how the disease is produced.

**Rickets is caused** by the necessary elements of albumen and fat being absent from the food, and by feeding children on starchy foods and skimmed milk. It hardly ever occurs in suckled infants; but it is developed in babies brought up by hand, or during or after weaning. Insanitary conditions, such as bad air and unwholesome dwellings, may aid in the development of rickets, but they are not sufficient to produce it; while food deficient in albumen and fat will cause rickets, even when the hygenic conditions are the very best.

**Infants Natural Food.**—I must stop for a moment to consider the proper and natural food for infants. This is, of course, mother's milk; but if this is not obtainable either from its own or a foster mother, then cow's or goat's milk, treated so as to resemble human milk. Now milk is a typical food, inasmuch as it contains all the elements necessary for nutrition, namely, albumen or nitrogenous matter, fat, a carbohydrate in the form of sugar of milk, salts, and water. The albumen is chiefly in the form of casien, which can be precipitated or thrown down from the milk by an acid or rennet, and it is thus that cheese is made from milk. The fat is suspended in the milk as minute globules, which can be clearly seen under the microscope. These globules of fat being light rise in the form of cream when milk is left standing some time. Skimmed milk is thus milk deprived of its fat, though as its albumen remains it is still a highly nutritious food for adults, but it is inadmissible for infants The sugar in milk is called lactose. Unlike ordinary sugar it cannot cause alcoholic fermentation. Thus animal milk remains as teetotal a beverage as water. The mineral salts, though small in amount, are of great value from a dietetic point of view.

To know **How to Feed an Infant Properly** when human milk is not available, it is necessary to know the nutritive value of other kinds of food, and to ascertain if they contain, in the proper proportions, the four necessary elements, namely, albumen, fats, carbo-hydrates, and mineral salts.

The following table gives the comparative analysis of the various kinds of milk:—

| Elements. | Human Milk. | Cow's Milk. | Asses' Milk. | Goat's Milk. | Artificl. Human Milk. |
|---|---|---|---|---|---|
| Nitrogenous or albuminous elements............... | 2·35 | 4·374 | 1·7 | 4·5 | 2·57 |
| Hydrocarbon fat........... | 2·41 | 3.499 | 1·4 | 4·1 | 4·46 |
| Carbo-hydrate or sugar of milk ..................... | 6·39 | 4·403 | 6·4 | 5·8 | 5·02 |
| Mineral salts.................. | ·34 | ·702 | — | — | ·57 |
| Water.............................. | 88·51 | 87·132 | 90·5 | 85·6 | 87·38 |
| Total .............. | 100·0 | 100·0 | 100·0 | 100·0 | 100·0 |

From this we see that cow's milk is richer in nitrogenous elements and fat than human milk, so that to make it suitable for infants it must be properly diluted; goat's milk is also richer, but asses' milk is much poorer than human milk. Now compare these perfect foods with the materials frequently given hand-fed infants, and which are productive of rickets. To do so the following table should be studied:—

| Food. | Albumen. | Fat. | Starch. | Sugar. | Salts. | Water. |
|---|---|---|---|---|---|---|
| Arrowroot ...... | — | — | 82·0 | — | — | 18 |
| Bread ............ | 8·1 | 1·6 | 47·4 | 3·6 | 2·3 | 37 |
| Wheat flour ...... | 10·8 | 2·0 | 6·3 | 4·2 | 1·7 | 15 |
| Oatmeal ......... | 12·6 | 5·6 | 58·4 | 5·4 | 3·0 | 15 |
| Biscuit ............ | 15·6 | 1·3 | 73·4 | | 1·7 | 8 |
| Cornflour ......... | 11·1 | 8·1 | 65·1 | | 1·7 | 14 |

It will be apparent on comparing this table with the previous one giving the various milk analyses, that the farinaceous foods which are given children on weaning and frequently to hand-fed infants, are deficient in fat and contain a superabundance of starch. This deficiency may be made good by the addition of milk, or still better, of cream to the bread, flour, or biscuit used. Obvious as may appear the teachings of nature, it is a fact that finding cow's milk disagree with an infant, some mothers, not knowing how it should be diluted and treated to make it digestible, will proceed to feed their babies on biscuits soaked in water, on arrowroot, on bread and skimmed milk, or on patent foods mixed with water, with the inevitable result of producing rickets. The fat and the albumen necessary for nutrition are withheld while the child is fed, or rather starved, on starch. Now starch, it will be observed, has no place in milk, and it forms moreover no element in an infants proper dietary, inasmuch as infants have in the early stage of existence no power of digesting or assimilating starch. Dr. Cheadle in his admirable book on the "Artificial Feeding of Infants" gives an instructive case of the production of rickets by deficient food in children of ignorant and well-to-do parents. The parents were prosperous tradespeople, but the mother was too much occupied with business to suckle or attend to her children. Of five children born healthy three had died in infancy. The child Dr. Cheadle was called to see was eleven months old, and had all the signs of well marked rickets. The symptom which had however alarmed the parents was spasm of the glottis, so severe as to threaten suffocation. On inquiry it was found that all the children had been hand fed on a patent farinaceous food, cornflour, and arrowroot made without milk, cow's milk having disagreed. Thus these little children were starved to death though abundantly fed. A proper dietary effected a cure in the case of the infant still alive.

**Symptoms of Rickets.**—The earliest and least distinctive symptoms of rickets are restlessness and slight feverishness at night; the child sweats profusely, and continually throws off its bedclothes. Next is noticed an unwillingness on the part of the child to be touched or moved; it seems sore all over, and has no longer any pleasure in being tossed about and caressed. The first positive evidence of rickets is given, however, by enlargement of the bones of the wrist and subsequently of the ankle, knee, and elbow joints. Then the long bones become bent and bowed, the ribs fall in laterally, their ends form knob-like projections, and the sternum projects in front, causing the well-known pigeon breast; the bones of the head are thickened, and the fontanelles remain open long after the time they are closed in healthy children; the head becomes large, flat on the top, with projecting forehead; the teeth are late in appearing. While these deformities in the bony skeleton are taking place, the general condition grows worse, fever increases, perspirations are more profuse, and the tenderness of the body becomes so great that the child dreads being touched. Appetite fails, weakness increases, the child emaciates and has a wan, anxious, pallid look. The abdomen protrudes and the liver and spleen are often found to be hypertrophied. When rickets prove fatal, death is caused either by lung trouble induced by the falling in of the thoracic walls, or by impaired digestion and consequent weakness, or by croup or convulsions.

Rickets may exist in a much less marked degree; the ends of the long bones may be thickened, but the constitutional symptoms may not be so marked. Recovery is the rule; and persons who have suffered from rickets in their youth may become very strong, but they are usually short, and the deformities of the bones, the bow legs, the curved spine, and the narrow chest generally remain, and often cause much misery and discomfort in after life.

**If the Bones be Examined** in rickets, it is found that in the cartilaginous extremities, where growth is most active, there is considerable enlargement, softening, and rarifaction, and that the earthy matter present is much less than in the bones of healthy children. It was hence formerly argued from this fact that rickets was caused by the want of lime salts, and that to give a child lime water and phosphate of lime would cure the rickets. This is, however, quite insufficient; many of the farinaceous foods on which ricketty children are fed are, moreover, rich in lime and phosphoric acid. Inasmuch as the disease of rickets is caused by food deficient in fat and albumen, so the cure of rickets lies in restoring these elements to the diet of the child.

**Dietetic Treatment.**—Dr. Cheadle considers that too much reliance is placed on cod-liver oil, chemical food, lime, and iron. Drugs are not so useful as dieting. Cream can take the place of cod-liver oil, milk of chemical food, and albuminous foods of iron. The diet of a ricketty child should be most carefully examined, and when it is found, as it usually is, that the child is being fed too exclusively on a farinaceous food, the missing elements of fat and albumen must be restored. This is best done by means of cream and cod-liver oil. If cream cannot be taken, boiled cow's milk, milk puddings of entire wheat, and raw meat pulp (the making of which was described in the chapter on invalid foods). Syrup of lacto phosphate of lime is useful; but the best of all medicines are fresh air, sunlight, and an outdoor life. Parents should be on their guard with respect to many of the condensed milks advertised as good and reliable foods for infants. As has been recently pointed out in the *British Medical Journal*, some of the advertised condensed milks are made of separated milk deprived of 90 per cent. of their fats, and are worse foods for infants even than skimmed milk. To bring up an infant on condensed separated milk is to ensure its having rickets. Those who have the care of children will be glad to know that the Milkmaid Brand of Anglo-Swiss Condensed Milk is reliable, and is made of whole milk; but mothers must be not only wise but wary if they would have healthy and happy children.

### The Pride of Mile End.

We are told that Mile End Guardians are proud of two results of their public policy. We certainly join with them in admiring the spirit of progress which resulted in the training of probationers at their infirmary in the very early days of the movement, and we can certainly congratulate them that the infant mortality in the district is a small one. There has been a marked neglect of the vaccination regulations, and the many thousands of unvaccinated it is suggested for the strength of the infant population of Mile End. We would rather inquire for the name of the officer of health for the district and look for a class of judicious mothers who have obeyed the laws of health in the care of the children if they have at the same time disregarded those of vaccination.

### Minor Appointments.

Miss Elizabeth Davis has been appointed Night Superintendent, in place of Miss Lofts, at the Chelsea Infirmary. She was trained at Chelsea, and has held the post of Charge Nurse there for the last three years.

Miss Emmie Lofts has been appointed Assistant Matron, in place of Mrs. Warren, at the Chelsea Infirmary. She was trained at St. Bartholomew's Hospital, and held the post of Night Superintendent at Chelsea until the present time.

Miss Shortrede has been appointed Assistant Matron at the Sick Children's Hospital, Edinburgh. She was trained in this institution, and was for four years in the Victoria Infirmary, Glasgow.

### Presentation.

On August 31st, at the City of London Hospital, Miss A. E. Morton was presented by the house staff and pupils with a handsome dressing-case in acknowledgment of the courtesy and energy with which she had filled the post of staff midwife during her year of office.

### Notes and Queries.

### Queries.

(134) *The Laundry.*—A Nurse wishes for directions how to get up collars and cuffs with a glaze and without.

(135) *Cancer Hospitals.*—(1) What homes or hospitals are accessible to female persons suffering with cancer, either by payment or without? (2) Mode of application necessary.—*W. E. M.*

(136) *Stewardess.*—Can you tell me how to obtain post as stewardess of a sailing vessel, and which lines employ only certificated nurses?—*M. L. W.*

(137) *Sick Nurse.*—Please tell me of a convalescent home where a hospital nurse can be received free of charge.—*M. V. H.*

### Answers.

(134) *The Laundry (A Nurse).*—After cuffs and collars have been washed and dried, they should be put through cold water starch, to which a little borax has been added, to prevent the iron sticking. Wring them out tight and roll them in a dry cloth till required for ironing. Iron quickly till quite stiff. If a glaze is required, it will then be necessary to use a glossing iron, which must be worked evenly with a rotary motion over the surface till the required amount of glaze is produced. Before commencing to glaze, rub the part to be covered by the glossing iron with a damp piece of cloth.

(135) *Cancer Hospitals (W. E. M.)*—Patients are received free on application, clothing and washing paid by patient, at the Cancer Hospital, Fulham Road, Brompton; into the Middlesex Hospital Cancer Wards by subscriber's or governor's letter.

(136) *Stewardess (M. L. W.)*—We have already treated this subject several times in The Hospital. Write to any shipping company—the addresses are in daily papers—and apply for form of application to fill in.

(137) *Sick Nurse (M. V. H.)*—If you can obtain no admission to a Home through other means, you can apply to "The Hospital Convalescent Fund" through the Editor, if the nurse is convalescent.

☞ For Character Sketches from a Lunatic Asylum, Everybody's Opinion, Novelties for Nurses, &c., see p. ccxxxi. et seq.

### Character Sketches from a Lunatic Asylum.

### THE PATIENT WHOSE LANGUAGE WAS UNINTELLIGIBLE.

She arrived one evening after dark, and entered the place with a stately and majestic air as if she were a queen taking possession of her kingdom. She was a lady of colour—a very black one indeed—and rather an old lady, too; for the crisp little curls of wool on her head were of a rusty black, inclining to grey—a sure sign of age in a South African native.

She was clothed in a skin only, which, on examination, proved to be a really beautiful piece of work. It was not, in fact, one skin, but several small skins of wild cats and such animals carefully prepared and sewn together with the sinews of oxen in that neat and dexterous manner characteristic of the natives. This was her garment by day, and her bed at night; but as these free and easy customs cannot be altogether countenanced in a Government institution, she was presented the next morning with a civilized garment in the shape of a dress, which she did not in the least know how to put on. It was a work of time and of persuasion to get her into it; and when it was at last accomplished, she did not seem particularly pleased with the result.

Our friend was a Basuto woman (so I was told) and as no one in the place could understand a word she said, not even the other native patients, who were mostly Kaffirs, it was impossible to discover what her mania might be, or whether she had any delusions. However, it became evident, after a while, from her impassioned conversations with empty space, that she heard voices speaking to her, and that the remarks they made were mostly of an aggravating, not to say, an insulting nature.

She soon proved herself to be an old woman of marked individuality. In many respects she was more like a man than a woman. She had a deep, gruff voice, was very strong and muscular, and had been accustomed, as is the manner of her tribe, to work in the fields while the man did the house work, of which she was quite incapable. She was extremely willing, even anxious, to do work of some kind, and one of the nurses, taking advantage of this disposition, tried to teach her to scrub. But it was hopeless. To begin with, she refused to kneel down to it, but persisted in standing, and stooping down — a position in which no one can scrub a floor, with the best intentions; and in the end, her only idea was to swamp the room with water, and then leave it to dry up at leisure. She subsequently retired on to the "stoep" or verandah with the bucket of water, and began to wash the doors and walls outside, throwing handfuls of water against them, and letting it run down until the whole place was deluged, and her well-meant efforts had to be checked. Another day she was tried with sewing, and if it had been a sail or a pair of leather breeches that she was called upon to make her work might have passed muster very well, but very fine needlework was evidently not her province. Finally, she was instructed in the art of washing clothes, and having a passion for water she took to it very kindly, and made herself useful in the laundry.

She was a very clean old woman indeed. Every morning as soon as she was let out of her room she went straight into the courtyard, where the grass was long and heavy with dew, and washed herself in the dew from the crown of her woolly head to the soles of her feet. Nothing made her more angry —and she had a violent temper—than to see one of the other patients throw dirt on the clean stoep, or anything of that sort. She would turn upon them at once, and peace-makers had to intervene.

She took a violent dislike from the first to two people in the institution—the matron and the head laundress—and was really dangerous when either of them came in sight, running to pick up stones wherever she could find them and throwing them at the objects of her aversion. After some time she forgot this feeling as regarded the laundress, to whom she became attached when she worked in the laundry; but she always preserved a distant and haughty demeanour towards the matron, though she left off active hostilities.

On the other hand, she had a great fancy for one of the nurses, who had taken the most pains in teaching her to work, or rather, trying to teach her. She would run to carry buckets for her, and make patient efforts to do anything she asked her to do, and in some mysterious manner she discovered where her bed-room was; for when the nurse was away on leave for a fortnight the old lady made her way, somehow, every morning to her bed-room door, and kicked it until she had to be borne off by main force.

As a rule, her wrinkled old face bore a rather melancholy expression, but every now and then she had an excess of high spirits, when she would dance, after her own fashion, which was not that of the Kaffirs, and sing in a monotonous and (to unaccustomed ears) a heart-rending chant. As her lungs were extremely powerful, and she liked to exert them to the utmost, when she began to sing no other sound was audible. This propensity was inconvenient at night, when she frequently lifted up her voice in the dormitory, at about three o'clock in the morning, to the great discomfort of the other patients.

To get her to take medicine of any kind was a herculean task indeed. Fortunately, she seldom, if ever, required it, being in robust health. She was moderate in eating, but sometimes she would regale herself with plaster off the walls which she broke off with her teeth, and eat with apparent relish. She also enjoyed a piece of coal if it came in her way, while her chief recreation was to grind a piece of tobacco, if she could get it, between two stones, preparatory to chewing it. These, however, were no symptoms of insanity. but habits common to all the natives.

On one occasion she was missed, and finally discovered in the upstairs room appropriated to the better class of European patients, where she was sitting quite quietly on the floor, close against the piano, listening to one of the patients playing. The sound of a concertina excited her greatly, and sent her off dancing and singing vigorously.

It was a great drawback to the understanding of her case that her language was unintelligible. She herself soon picked up a few words of Dutch from the other patients, but not enough to express herself clearly, and her delusions (for there was no doubt that she had delusions) remained a mystery. She would stand up on any elevated place that she could find, and hold forth by the hour, working herself into quite a passion with her imaginary opponents, and declaiming apparently with great eloquence, but the subject of her discourses no one ever knew.

She was very independent in her habits, and troubled herself very little about the other patients, or indeed about anyone, as long as they did not interfere with her.

When she first came she used to try and escape, more than once breaking a window, and being caught half-way through it; and when prevented in her attempt, the tears would sometimes run down her withered old cheeks, making one quite sad as one wondered what confused memories of her home and her free wild life might be stirring in her breast. No doubt she had become unmanageable and dangerous among her own people, yet she had a willing, affectionate disposition, and one could not help growing quite fond of the poor old soul. Latterly she settled down and seemed to become more reconciled to her new home and mode of life. Probably she gradually forgot her former life and her own people, and lived, more happily for herself, entirely in the present.

A. H.

### Everybody's Opinion.

[Correspondence on all subjects is invited, but we cannot in any way be responsible for the opinions expressed by our correspondents. No communications can be entertained if the name and address of the correspondent is not given, or unless one side of the paper only be written on.]

### ABUSE OF UNIFORM.

"Nurse M." writes: I have been greatly annoyed several times, and in two different towns (Sheffield and Derby), by seeing women dressed up as hospital nurses walking the streets selling patent foods. The behaviour of these women I consider simply disgraceful. If our uniform is to be used in this way, and by such women, can we expect it to be any protection? I should like to know if anything can be done to put a stop to this kind of thing.

### BATHS.

"A Trained Nurse" writes: Your correspondent (A. F. Jones) has started a subject upon which I know there is some difference of opinion—cold or tepid baths when hot after exercise? I am strongly in favour of the tepid bath, because I think to take a cold bath immediately after exercise, when the surface of the skin is hot and the pores are open, tends to check the natural action of the skin. I have known of more than one serious illness, and, in two cases, of a skin eruption caused by taking a cold bath when hot after exercise.

### MONTREUX.

"Nurse M. G." writes: Could you give me any information through your columns about private nursing in Montreux? I have been told that English nurses are greatly in demand there, and I should be very glad to have some reliable information on the subject if possible. Perhaps some Hospital reader could advise me as to the proper person to apply to. I shall be exceedingly grateful for any information. [See "The Nursing Mirror," for June 2, 1894, p. lxxxvi., "Nursing in Switzerland."—Ed. *T. H.*]

### THE POSITION OF MATRON IN SMALL FEVER HOSPITALS.

"Perplexed" writes: The position of a matron in the smaller provincial fever hospitals is often anything but a happy one, and, unfortunately, these appointments are often accepted by those unaware of what they will have to contend with. I refer more particularly to the pernicious system of the nurses being engaged by the committee; the nurse knows in consequence that the matron has no power to dismiss her, and there are many little matters too trivial to report to a committee, but which the matron could rectify if she were invested with the authority which she ought to have. I believe that, generally, a committee, having proof that confidence may be placed in the matron they appoint, if approached on the subject would, be willing to alter this undesirable state of affairs "in the future." But what is the onsequence? If possible, it makes bad worse, for, as any change occurs or an increase is needed, the matter is left to the matron, and, in consequence, she has a part of the staff entirely under her own control and a part not; the latter portion are very apt to create dissatisfaction should any little unpleasantness occur (and where will it not?) by rejoicing in the fact that they were engaged by the committee and not by the matron. If nurses knowing this would make a stand against such a system, and refuse to accept an appointment where they are not invested with the entire control of the staff, boards of management would soon be brought to see that in order to obtain the services of a competent matron they must give her absolute authority, as much as the entire responsibility which is always thrown upon her, and which is no sinecure in a hospital with no resident medical officer.

### Death in our Ranks.

It is with much regret that we record the death of Henrietta Mary Louisa Coulton, which took place at Pentney, Norfolk. Miss Coulton was much respected by her fellow workers at the London Hospital, where she several years ago received three years' training.

### Novelties for Nurses.

### WOOLLEN WINTER GARMENTS AND MATERIALS.

(Messrs. Fleming, Reid, and Co., The Worsted Mills, Greenock.)

We can always speak with unqualified praise of Messrs. Fleming, Reid's knitted underclothing. Having ourselves worn their garments for some time, we can from experience testify to the fact that in durability they compare most favourably with other purchases we have made of the same kind, and that their shape and finish is superior to any we have yet come across at the same prices. The price of all the garments is very moderate, and although cheaper articles may be purchased, we have found ourselves they are in the long run more expensive. Nurses will do well if they send for a catalogue from Messrs. Fleming, Reid before purchasing warm winter clothing, stockings included. We do not know whether the firm supplies patterns of various knitted wools of which the underclothing is made, but it would be as well to enquire, as our inspection of such samples would prove to intending purchasers that we have not been too lavish in our praise. Besides manufactured goods Messrs. Fleming, Reid supply excellent knitting wools, and of these we know they have patterns. The prices of these vary from 1½d. the oz. for 4 ply Scotch fingering, to 3d. the oz. for double Berlin.

We should like also to draw the attention of our readers to the excellent quality of the woollen materials manufactured by Messrs. Fleming and Reid. A beautifully fine cloaking commends itself especially to our notice at 2s. per yard, 46 inches wide, and another in a stouter make, intended to defy all weathers, is not expensive at 5s. 6d., and is 56 inches wide. The diagonal serges are very soft and pretty, and as they are composed of pure wool, will be found most durable. A fine black foulé which this firm is now showing, is particularly well adapted for summer wear and is equally reasonable as regards price. These desirable goods are to be had in all qualities and colours and intending purchasers cannot do better than send for samples.

### PLATINUM DRESS BONES.

(Messrs. Herts and Co.)

Messrs. Herts, whose excellent speciality the Ant Platinum Corset we have already noticed recently, now send us specimens of their platinum bones. These possess considerable advantages; they are less expensive and more durable than whalebone, they do not rust, are more elastic, stronger and more durable also than steel, hitherto the only substitute in dressmaking for whalebone. The platinum bones can be had in different lengths and thickness, and covered in cotton or silk to suit the purchaser.

### A Case for Assistance.

Our appeal for the disabled Matron has resulted in a further list of contributions, which we give below. We hope that our suggestion that Nurses and Matrons should start a subscription list in their institutions will provide the £7 yet needed to secure the little business which will render the Matron for whom we have pleaded independent. Miss Bell, Home for Aged Mariners, Egremont, Cheshire, will gladly receive contributions at once, as we cannot keep the fund open longer than next week. List of contributions since August 27th: Nurse C. M. (Crumpsall), 3s.; Sister Hope (Guildford), 5s.; Nurse Maud (stamps), 6d.; Sister Rose (stamps), 1s.; Nurse A. W. H. and Sister (stamps), 1s.; F. E. G. (collected), (An old King's Nurse), 8s.; A. E. B. (Portsmouth), 5s.; J. M. Davis (West Brighton), 1s.; Sister M. (Tunbridge), 2s. 6d.; Nurse (Weston-super-Mare), 1s.; Nurse Holland, £1; Nurse Geldert, 1s.; C. H. (second donation), 3s.; Miss Buchanan, 5s.; A Lover of Justice, 4s. Total to September 3rd, £13 1s.

### The Book World for Women and Nurses.

[We invite Correspondence, Criticism, Enquiries, and Notes on Books likely to interest Women and Nurses. Address, Editor, The Hospital (Nurses' Book World), 428, Strand, W.C.]

"A Wasted Crime." By David Christie Murray. (London: Chatto and Windus.) Two vols., 1893.

From the title of the story the reader will be prepared for blood and thunder, and the sequel proves that his anticipations are well founded. The author's name, the reader would also expect, would be a sufficient guarantee of literary accuracy, if not of literary merit. In this respect he will be disappointed, and if critically inclined, or wise in matters medical, he will find several weak spots on which to focus his criticism. The plot of the story may be summarised as follows. Robert Audley, the scion of a noble house, falls in love with and finally marries a school teacher of humble birth, but of aspiration and ambition of the penny shocker description. Sir William Audley, père, becomes furious and threatens to cut Robert off with a shilling; in fact he draws up a will to this effect, but with the accommodating good luck peculiar to novels he is prevented from executing the same by a timely accident which lays him helpless on his back. Now comes Mrs. Robert's opportunity. She personates a devoted nurse, wins the confidence of the old man, discovers that the will has not been executed, and sees the advantage of accelerating the baronet's departure from this world by an overdose of physic. Subsequently she discovers that the old man must have died in any case, and hence the "wasted crime." Her crime being discovered, the heroine finds relief in a six ounce bottle of chloral. It is usual for an author to make himself acquainted with the technicalities of the subject he deals with, even though the book be a novel, and the subject medicine. Readers of The Hospital will be amused at the shortcomings in the latter respect. The accident which befell Sir William Audley necessitates the sending for a specialist from town. In a case which was eminently surgical, why call in the services of an "eminent physician?" and why later call the eminent specialist a "practitioner"? and surely the great man talks nonsense when he says: "You have had a shock, Audley! You consulted me twenty years ago, and I never forget a patient. That aneurism of the heart has always been your danger." Repeated blemishes of this description relegate the book to a position amongst second-class novels. It is, however, a book that will agreeably while away a few hours.

### MAGAZINES OF THE MONTH.

The English Illustrated Magazine for September contains a notice that the following month's issue will prove itself "the most readable and the most artistic of monthlies and will in many respects be a new departure in magazine literature." In this scheme no serial stories or continued articles will find a place, and this, we must remark, is a step in the right direction; the public are gradually and surely discovering the difficulties of keeping in their minds the thread of a narrative in the intervals of serial publication. But the September number is in itself an earnest, so to speak, of the better things we are led to anticipate. Here, with the exception of "Some Jewel Robberies," no article is "to be continued in our next." The short and complete story by Barry Pain is a striking contribution, and is both strongly and humorously written.

In September's Cornhill, "A Fatal Reservation," continues to run its course. James Payn gives us his third "Gleam of Memory." Our interest continues to be sustained in "Mathew Austin" (W. E. Norris). A particularly bright sketch is "The Shadow on the Blind," which is unsigned. The history of a retired business man's occupation in his hunt for new homes is both graphic and to the point. "This hunting for homes, and the letting them go that he might pursue game of the same kind elsewhere, was naturally more entertaining to Mr. Stackpoole than it could be to his wife and daughter," and the descriptions of this are extremely amusing.

The Leisure Hour contains much matter of an interesting nature. We always learn through these pages many things regarding men and matters—the present issue has proved no exception to the rule. "The Observatory on the Summit of Ben Nevis," perhaps, is the most attractive among these pages. It describes both the work and the life of the "martyrs to science," as Mr. Whymper puts it, of the dwellers on this height. The work, alas, is a heavy one, owing to a limited staff, and a touching appeal is made by the writer for pecuniary aid for an extended help, from those who have money to spare among us and who could thus render something towards a useful and deserving public service.

### For Reading to the Sick.

### THE GOOD SHEPHERD.

**Verses.**

I was a wandering sheep,
I did not love the fold,
I did not love my Saviour's voice,
I would not be controlled.
I was a wayward child,
I did not love my home,
I did not love my Father's voice
I loved afar to roam.

The Shepherd sought His sheep,
The Father sought His child,
They followed me o'er vale and hill,
O'er deserts waste and wild;
They found me nigh to death,
Famished and faint and lone;
They bound me with the bonds of love.
They saved the wondering oue.

They spoke in tender love,
They raised my drooping head,
They gently closed my bleeding wounds.
My fainting soul they fed.
They washed my filth away,
They made me clean and fair;
They brought me to my home in peace,
The long-sought wanderer.

—*Horatius Bonar.*

**Motto.**

The Lord is my Shepherd, therefore shall I lack nothing.

—*Psalms.*

**Reading.**

. . . The Good Shepherd has called me, but I did not hearken to His voice; I wandered on farther from the fold and from Him, farther into carelessness and worldliness; . . with little thought of the Loving Shepherd. But He did not leave me alone. He went after me seeking diligently, and then He put forth His hand and laid it on me.

Ah, His hand was heavy at first, so it seemed to me. But it was needful, it was in love. He could not draw me back, He could not lift me out of the brambles and mire without hurting me a little. Without a little sharp pain, even He could not bring me back into the paths of peace.

I understand now why this illness came to me. It made a pause in my life, a pause that I did not like, a painful stop in the midst of my blind, easy wanderings.

But I am forgiven. The Good Shepherd has given His life for the sin of the sheep. He has died to win me back.

O wondrous Love! How can I do aught else but pour out before Thee all the poor love of my heart!

"For ye were as sheep going astray, but are now returned unto the Shepherd and Bishop of your souls."

—*Caroline M. Hallett.*

